# Finding retrieval-induced forgetting in recognition tests: a case for baseline memory strength

**DOI:** 10.3389/fpsyg.2014.01102

**Published:** 2014-09-29

**Authors:** Bernhard Spitzer

**Affiliations:** Neurocomputation and Neuroimaging Unit, Department of Education and Psychology, Freie Universität BerlinBerlin, Germany

**Keywords:** retrieval-induced forgetting, recognition, signal detection, recollection, familiarity

## Abstract

Retrieval practice of previously studied material can impair subsequent memory for related unpracticed material. An emerging view holds that such retrieval-induced forgetting (RIF) may affect episodic recollection, but not the context-free familiarity of the affected items. Here, a survey of accruing recent findings of RIF in recognition tests shows that the impairment of unpracticed material depends vitally on baseline memory strength. Therein, the absence of RIF under specific conditions, previously taken as evidence for the immunity of familiarity, can be predicted on grounds of exceedingly low baseline levels. Similarly, differential RIF effects on the parameters of dual-process recognition models can be explained by baseline differences, suggesting that RIF might impair any sub-process that substantially contributes to overall recognition accuracy. By contrast, the strengthening of practiced material appears independent of baseline levels and does not predict the magnitude of RIF, in accordance with an inhibitory causation of the forgetting. In summary, the inventory presents RIF in recognition as a subtle proportional impairment, future illumination of which may demand increased attention to baseline memory levels.

## REMEMBERING CAN CAUSE FORGETTING

Retrieval-induced forgetting (RIF) refers to the finding that retrieval practice of a subset of previously studied items (RP+, for instance, Fruit Or___) may impair later memory for related unpracticed material (RP-, e.g., Apple). Even 20 years since the seminal demonstration of this phenomenon (Anderson et al., 1994), the precise mechanistic causes remain subject to debate. One prominent account holds that during retrieval practice of RP+, interfering RP- items are suppressed and become enduringly inhibited (e.g., Anderson and Spellman, 1995), resulting in impaired memory performance on subsequent tests, compared to unrelated control material (NRP). While supported by a wealth of experimental evidence (for review, see, e.g. Storm and Levy, 2012), the inhibition account remains disputed in favor of alternative, non-inhibitory explanations, most of which assume that the RP- impairment occurs only at the stage of final testing, for instance due to pervasive interference of the strengthened RP+ items (e.g., Raaijmakers and Jakab, 2012; cf. Jonker et al., 2013).

One important aspect in the discussion has for long been whether RIF occurs only in recall tests, where the phenomenon was routinely demonstrated, or also in tests of recognition memory. *A priori*, impaired recognition may be expected if, as suggested by inhibition accounts, suppression during retrieval practice weakened the RP- materials’ memory representation *per se*, which may manifest in any subsequent test of memory (for in-depth theoretical considerations, see Anderson, 2003). Experimental support for this view was provided by Hicks and Starns (2004), who reported reliable RIF impairment of RP- in a conventional item recognition test. The effect replicated in a second recognition experiment, where the impairment was further found to generalize to source memory judgments, suggesting that inhibitory weakening may pull down not only item-specific, but also item-context associative memories, in good agreement with the routine finding of RIF in recall tests.

## RECOLLECTION, FAMILIARITY, OR BOTH?

According to many neurocognitive concepts of episodic memory, recognition can be decomposed into at least two distinct sub-processes: an immediate feeling of context-free “familiarity,” and/or the recall-like “recollection” of episodic details of the study context (for review, e.g., Yonelinas, 2002; Sadeh et al., 2014). From such dual-process (2P) perspective, the findings in item recognition and source memory by Hicks and Starns (2004) suggested that RIF may affect both processes. Soon, however, Verde (2004) reported conflicting evidence. In two experiments, RIF was found in item-item associative recognition, that is, a procedure assumed to particularly emphasize recollection. In contrast, no RIF occurred in a condition where the time to memorize the item pairs during initial study was shortened. Reasoning that shorter study times selectively reduced the contribution of recollection, the results were seen as evidence that RIF leaves familiarity unaffected.

We sought to further detail the specific effects of RIF by applying various formal models of recognition memory (Spitzer and Bäuml, 2007). In two experiments, we replicated Hicks and Starns’ (2004) finding of RIF in single-item recognition. In terms of 2P model parameters however, the impairment was attributed primarily to reduced familiarity, with less reliable reductions of recollection. The latter appeared surprising in light of the earlier literature, raising the question whether 2P modeling actually gave an adequate account of the data. Indeed, in our formal model comparisons, the available 2P models were outperformed by a ordinary (unequal-variance-)signal-detection (SDT) model (see also Wixted, 2007; Dede et al., 2014). In particular, the unidimensional SDT model superbly fitted not only our own but also Verde’s (2004) data, and appeared to resolve the previous contradiction in findings: In terms of SDT parameters, the RIF impairments in the different studies were all coherently characterized by a reduction in general memory strength *d*′ (Spitzer and Bäuml, 2007). It should be noted that such SDT-approach, albeit formally a “single-process” description, does not preclude the potential contribution of familiarity and/or recollection (or any other process) to recognition performance (Dede et al., 2014). Rather, conventional SDT models are agnostic as to whether different sub-processes can be decomposed and quantified from the meager trial-by-trial information (usually one data point) provided in behavioral recognition tests.

The question of recollection and/or familiarity was more recently addressed anew by Verde and Perfect (2011), who reported RIF to be absent under time pressure at test. More specifically, while one group of their subjects replicated the reduction of *d*′ in a standard item recognition test (Hicks and Starns, 2004; Spitzer and Bäuml, 2007), another group was required to respond (“old”/“new”) within less than 750 ms. Because no RIF was found in this group, and because speeded recognition is thought to prioritize familiarity over recollection, the results were taken as model-free evidence that RIF exclusively impairs recollection, but not familiarity or an item’s memory strength in general.

Taken together, previous studies into the nature of RIF in recognition yielded an inconsistent picture. Some results favor a selective disruption of contextual recollection, which might not necessarily be specific to inhibitory forgetting but could also be caused by RP+ pervasion at test. Other analyses suggest that RIF, in agreement with inhibitory suppression, may entail a more direct weakening of the affected item representations, and might be described as a reduction in general mnemonic strength, which may include not only recollection but also familiarity. The question arises how the discrepant experimental results behind these views might be integrated when seen in a broader context of accumulating recent findings of RIF in recognition tests.

## A SURVEY OF THE EVIDENCE

A search of the experimental literature since the report by Hicks and Starns (2004) yields more than 20 peer-reviewed studies of RIF with recognition testing, 10 of which were published only since 2011 (**Table 1**). Expectedly, the experiments covered a broad range of specific settings (e.g., special populations, emotional manipulations, or concurrent distracting tasks), such that in several cases, only the control conditions/groups were included in the present survey (see **Table 1**). A further requirement was the availability of standardized *d*′ measures (resp. data required for their computation), for comparability across experiments. In total, 28 experiments/conditions from 20 studies were included.

**Table 1:** Studies reporting RIF in recognition memory.

	Study/condition	Recognition test		Study/condition	Recognition test
*1a*	Hicks and Starns (2004)* Experiment 1	Item	*11a*	Verde and Perfect (2011) condition “self-paced”	Item
*1b*	Experiment 2	Item + source	*11b*	condition “speeded”	Item, speeded
*2a*	Verde (2004) Experiment 1	Item–item associative, RK	*12*	Aslan and Bäuml (2011)	Item
*2b*	Experiment 2, condition “long study”	Item–item associative, RK	*13*	Ortega et al. (2012)* Experiment 1, young adults	Item
*2c*	Experiment 2, condition “short study”	Item–item associative, RK	*14*	Aslan and Bäuml (2012) Group “young-olds”	Item
*3*	Gómez-Ariza et al. (2005) Experiment 2	Sentence	*15a*	Ortega-Castro and Vadillo (2013)*, condition “A-B, A-C”	Item
*4a*	Spitzer and Bäuml (2007) Experiment 1	Item, RK	*15b*	condition “A-B, C-B”	Item
*4b*	Experiment 2	Item, rating	*16*	Janczyk and Wühr (2012)* Experiment 1a, condition “standard”	Item
*5*	Dehli and Brennen (2009)* condition “neutral emotion”	Item	*17a*	Dobler and Bäuml (2013) Experiment 2, condition “blocked” (rp)	Item, rating
*6*	Spitzer and Bäuml (2009)	Item–color category, rating	*17b*	Experiment 2, condition “mixed” (rp)	Item, rating
*7*	Soriano et al. (2009)* Experiment 2, healthy controls	Item	*18a*	Luna and Martín-Luengo (2014)* Experiment 1	Item + confidence
*8*	Román et al. (2009)* “Single task” control	Item	*18b*	Experiment 2	Item + confidence
*9*	Spitzer et al. (2009)*	Item	*19*	Erdman and Chan (2013) Experiment 2, “no feedback”	Item
*10*	Aslan and Bäuml (2010)* Adult control subjects	Item	*20*	Grundgeiger (2013) Experiment 1, “competitive”	Item

**FIGURE 1:**
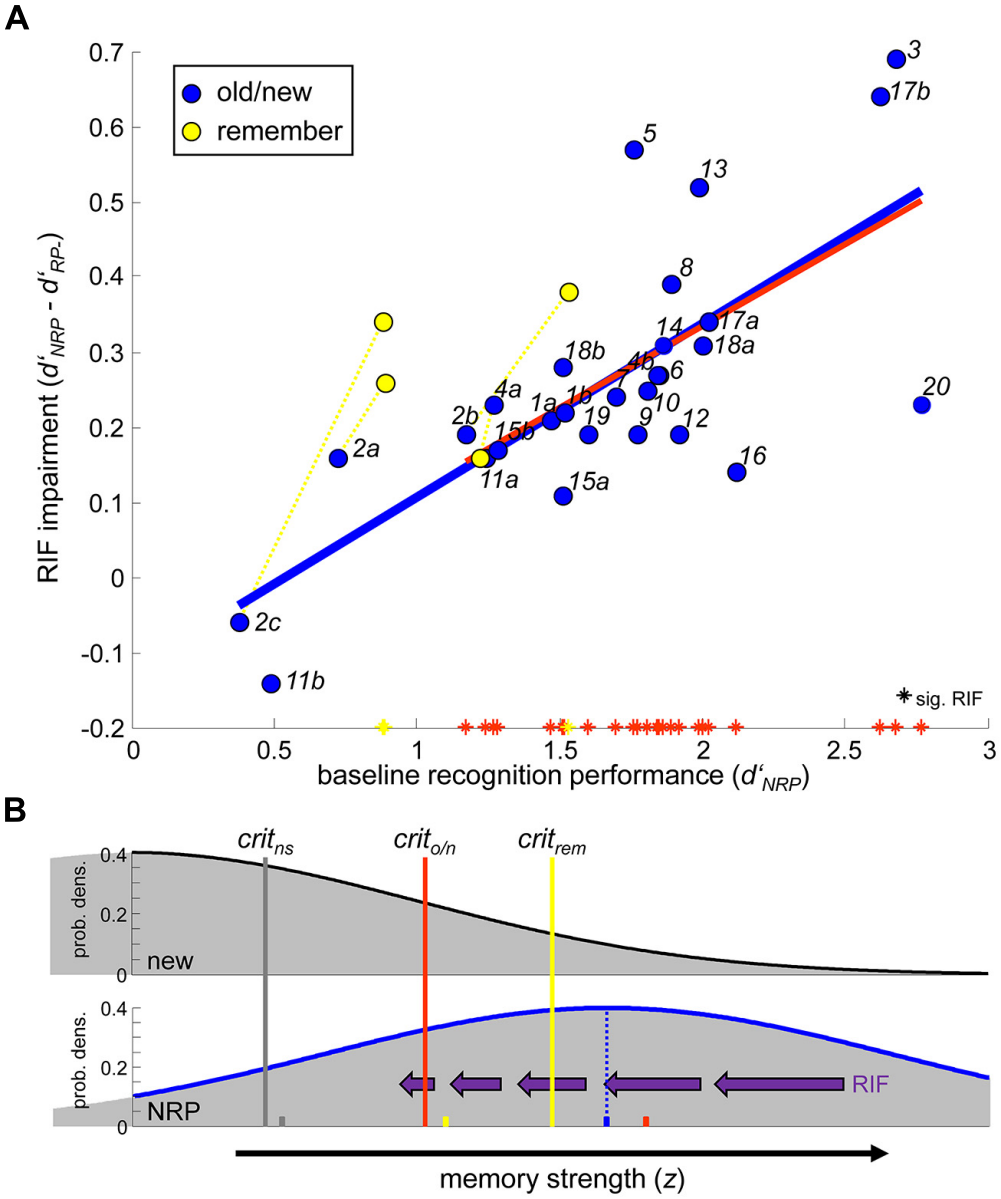
**(A)** Retrieval-induced forgetting (RIF) effects on recognition performance as a function of baseline-memory strength. *Blue dots:* Old/new recognition. Italic data labels denote study/condition reference (see **Table 1**). *Solid blue*: Linear fit. *Solid red*: Linear fit including only those conditions that exhibited a significant RIF impairment in old/new recognition (tagged with *red asterisks* on x-axis; includes all conditions with *d*′_NRP_ > 1). *Yellow dots:* “Remember” recognition in the conditions referenced by *dashed yellow lines*. Significant “remember” RIF is tagged with *yellow asterisks* on x-axis. **(B)** *Upper*: Gaussian “noise” distribution according to SDT [φ(*z*), standard normal] for unstudied new material, relative to which baseline memory strength (*d*′_NRP_) is assessed. *Lower*: Gaussian distribution of baseline (NRP) memory strength in a prototypical RIF experiment (μ *= d*′_NRP_ = 1.67) according to SDT. Purple arrows symbolize the hypothesized trial-by-trial effect of RIF on memory signals of different strength, with effect sizes (∼arrow length) derived from the linear fit (*blue*) in (A). Vertical marker lines indicate location of the mean response criteria in conditions without significant RIF (“ns”; *2a,2c, and 11b*), in conditions with significant old/new RIF (“*o/n”*; see *red asterisks* in center figure), and in conditions with significant “remember” RIF (“*rem”*; see *yellow asterisks* in center figure). Same-colored markers on bottom indicate the grand average *d*′_NRP_ in the corresponding conditions.

Studies are listed in chronological order. In all cases, recognition testing involved old/new discrimination of previously studied material from new material. RK, remember/know judgments. Studies using “rating” procedures (*4b, 6, 17*) reported analyses of receiver-operating characteristics (ROCs). If available, *d*′ estimates (**Figure 1A**; see text) were taken directly from the studies’ results sections (for study *19* read from results Figure). For the remaining studies (marked*), *d*′ was computed from the reported hit- and false alarm rates (in 8 and 13 reconstructed from “corrected” hits).

**Figure 1A** illustrates the RIF-impairments of old/new recognition accuracy in the different experiments (*blue*; y-axis: *d*′_NRP_ *- d*′_RP-_). Notably, a significant impairment was found in each of the 20 studies, in at least one of the reported experiments/conditions (*red asterisks* in **Figure 1A**). This overall picture counters recent assessments that RIF in recognition tests might be observed only casually, and with little reliability (e.g., Jonker et al., 2013). At the same time, even when statistically reliable, the RIF impairments are typically not very large in size (average: *d*′ = 0.29; min: 0.11; max 0.69), which might explain singular mentions of unpublished failures finding such effect (Jonker et al., 2013; see also Koutstaal et al., 1999). It should further be noted that at least four additional studies demonstrated significant RIF in non-standard recognition measures (e.g., response latency; Veling and van Knippenberg, 2004; Racsmány et al., 2008; also see Saunders and MacLeod, 2002; Potts et al., 2012), which due to lacking comparability were not included in the present analysis. In its entity, the available evidence characterizes RIF in recognition as a subtle, but fairly well-replicated phenomenon.

## RIF IN RECOGNITION DEPENDS ON BASELINE MEMORY STRENGTH

The baseline accuracy levels (x-axis: *d*′_NRP_) varied considerably across studies (min: 0.38; max: 2.77). What is more, baseline performance appeared as a reliable predictor of the reported RIF effects (*solid blue* in **Figure 1A**; linear slope: 0.23; *r* = 0.73, *p* < 0.001). Therein, among the 28 conditions, those failing to find significant RIF in old/new recognition (*2a*, *2c*, and *11b;* reported in Verde, 2004; Verde and Perfect, 2011) stand out as those with the lowest baseline accuracy levels (all *d*′s < 0.75). However, a clear linear dependence was evident also when restricting the analysis to those data sets where significant RIF was observed (*solid red* in **Figure 1A**; linear slope: 0.22; *r* = 0.59, *p* < 0.002), and even when potential NRP outliers were excluded (1 < *d*′_NRP_ < 2.5: linear slope: 0.17; *r* = 0.42, *p* < 0.05). Of note, *d*′_NRP_ and *d*′_RP-_ itself were highly correlated (*r* = 0.96, *p* < 0.001), rendering it unlikely that the above pattern resulted only from biased sampling of independent random distributions. Overall, how much RIF was observed in a given experiment or condition appeared to depend on the “intact” baseline memory level.

One potential account for the above pattern might be in terms of selectively impaired recollection. Although *d*′ is a measure of continuous, strength-like signals (more akin to the traditional 2P-concept of familiarity than of recollection; see Yonelinas, 2002; Wixted, 2007), higher recognition accuracy may often come along with increased recollection, and thereby increased susceptibility to RIF. While plausible, this explanation is difficult to prove for the greater portion of studies finding RIF in standard recognition tests (see **Table 1**), which are thought to bring forward – albeit not exclusively – familiarity. Indeed, baseline-dependence was evident even when restricting the analysis to conventional single-word old/new recognition (*1,4,7-11a,12-20; r* = 0.45, *p* < 0.05). Of note, these standard tests appeared no less efficient in detecting RIF (mean: *d*′ = 0.28) than associative tests (*2a-b, 6;* mean: *d*′ = 0.24) or “remember” judgments (**Figure 1A** *yellow;* see below; mean *d*′ = 0.28) which are thought to prioritize recollection.

Alternatively, the baseline-dependence of RIF in recognition can be explained by the presumed nature of inhibitory suppression: During retrieval practice, stronger memories tend to have greater interference potential and are suppressed more strongly, resulting in greater inhibition (Bäuml, 1998; Anderson, 2003). In turn, little or no interference-suppression is expected for associations whose strength *a priori*, by study manipulation, hardly rose from the floor level of new (i.e., unstudied) material (e.g., in condition *2c*). The absence of RIF under such conditions, previously related to spared familiarity (Verde, 2004), may thus as well reflect a lack of inhibition for weak memories. A challenge for the possibility that inhibition may also affect familiarity might however be seen in the presence/absence of RIF under specific *testing* conditions. Before addressing these in detail, we may put the available data into the perspective of SDT.

## A SIGNAL DETECTION VIEW ON RIF IN RECOGNITION

Under the formal assumptions behind *d*′, NRP strength is the standardized mean distance (in *z*-units) from the mean of the “noise” distribution of new material, which is described by a standard Gaussian (i.e., μ*_new_* = 0, σ*_new_*= 1; **Figure 1B**, *upper*). Recognition performance (*d*′ > 0) stems from the differential probability for “true” (here: NRP), compared to “false” memory signals (here: new) to exceed a response criterion (*crit*∼*z*, “confidence,” see vertical lines in **Figure 1B**). The trial-by-trial distribution of NRP strength according to conventional SDT is exemplified in **Figure 1B** *lower* for a prototypical RIF experiment (*blue*, μ = 1.67, corresponding to the grand mean *d*′_NRP_ across all surveyed data sets). For simplicity, σ*_NRP_* is set to 1, although the variance of studied material is often found to be somewhat larger than σ*_new_* (for review, see Wixted, 2007). In the SDT framework, we can characterize RIF (*d*′_NRP_ - *d*′_RP-_) as a negative shift of RP*-* relative to the NRP distribution. Noting the trial-by-trial variability of NRP strength according to SDT, it seems likely that the baseline-strength dependence of RIF (**Figure 1A**) will be relevant also on the individual item level, such that stronger memories will suffer more from RIF (symbolized by purple arrows in **Figure 1B**, *lower*). In some respect, such adaptive strength reduction on a trial-by-trial basis shares similarities with the proposal of a selective disruption of recollection. However, whereas the latter view attributes RIF to a qualitatively separable class of “recollected” memories, the present SDT-account maps these as relatively strong memories onto one quantitative continuum, together with all other exemplars.

Evidence in favor of a selective disruption of recollection only was previously seen in the observation that RIF in associative recognition tests can be enhanced when focusing only on “remember” judgments (Verde, 2004), that is, test trials on which subjects introspectively reported the experience of recollection (Gardiner, 1988; see also Tulving, 1985). When this was the case, however (*2a-c*), “remember” judgments also tended to be more accurate (*yellow*) than old/new recognition (*blue*), corroborating that RIF may mirror baseline levels on a trial-by-trial basis (note also the consistently inverted pattern in *4a*). Further, ‘remember’ judgments are typically given with high confidence. In SDT terms, the mean “remember” response criterion in *2a-c* (**Figure 1B**, *yellow line*, *z* = 1.46) was actually higher than the mean response criterion in studies that showed regular old/new RIF effects (*red line, z* = 1.06), rendering “remember” judgments in *2a-c* particularly sensitive to reductions of relatively strong memory signals. Together, in light of SDT, the “remember” findings integrate well with the view that RIF predominantly affects stronger memories, which, on intact NRP baseline, would substantially exceed the noise level of new items.

A similar rationale may apply to the recently reported absence of RIF in speeded recognition (*11b*). At first, seen in the greater context of studies using standard recognition tests, the NRP performance level in *11b* was remarkably low (*d*′ = 0.49; note that such level can be reached even if 80% of the responses were pure guesses)^1^. Evidently, aside from its purpose of limiting recollection, the speeded testing condition picked up only little baseline memory strength overall (including familiarity). Yet, granted that detection of a RP*-* impairment would have been technically possible (Verde and Perfect, 2011), the absence of RIF under time pressure is not inconsistent with a possibly more general representational weakening under regular testing conditions: Translating the 2P-assumptions behind speeded recognition into SDT-terms, the manipulation may in particular limit stronger memory signals. In this respect, the effect of speeded instructions on intact baseline memory (cf. *11a-11b*) would be very similar in quality to the hypothesized effect of RIF itself, just more effective. Thus in light of strength-dependent inhibition, if a testing manipulation systematically deprives baseline performance of its diagnosticity for stronger memories, inability to detect RIF impairment may come as no surprise.

The above considerations do not preclude that for stronger memories, strength-dependent inhibition may proportionally decrease recollection. However, the SDT-perspective illustrates the difficulty of interpreting the available evidence with respect to potential (null-)effects on familiarity, clarification of which might prove essential for arriving at a conclusive 2P characterization of RIF in the future.

## RIF IN RECOGNITION IS INDEPENDENT OF RP+ STRENGTHENING

While various mechanistic accounts could in theory accommodate the baseline-dependence of RIF, non-inhibitory explanations may additionally predict a dependence on the strengthening of the practiced material (RP+), which is thought to interfere with recollection of RP- at test. In contrast, no such link between RIF and the benefits for RP+ is assumed in the concept of inhibitory suppression during retrieval practice (e.g., Anderson and Spellman, 1995). In accordance with the latter, the surveyed RIF impairments were not systematically related to RP+ strengthening^2^ (*d*′_RP+_ -*d*′_NRP(+)_; *r* = -0.29, *p* > 0.10), even less when excluding potential ceiling effects (*d*′_NRP(+)_ < 2.5 only: *r* = -0.02, *p* > 0.90). This result yields little support for a blocking of RP- recollection due to RP+ pervasion at test, which *a priori* could be expected to increase with the strengthening of the practiced material (for similar findings, see e.g., Hulbert et al., 2012). Further control analysis shows that the RP+ benefits were independent from NRP(+) baseline level (*r* = -0.19, *p* > 0.25; *d*′_NRP(+)_ < 2.5 only: *r* = 0.13, *p* > 0.50) suggesting that the baseline-dependence outlined in **Figure 1A** selectively concerns the detrimental effects of RIF, rather than reflecting unspecific differences in, e.g., global measurement variance between experiments.

## HOW WE FORGET MAY DEPEND ON HOW WE RECOGNIZE ON BASELINE

If baseline levels explain the magnitude of RIF in recognition, may they also account for the qualitatively inconsistent RIF-patterns seen in previous 2P analyses of recollection (*R*) and familiarity (*F*) parameters? Descriptively at least, the available modeling results strongly suggest this possibility. For instance, applying formal 2P models of remember/know responses (Yonelinas and Jacoby, 1995), we previously found the relative baseline contributions of *R* and *F* to associative recognition (Verde, 2004) to be on the order of 2:1, and RIF affected *R* about twice as much as *F* (for details, see Spitzer and Bäuml, 2007). In our own item recognition data, the baseline contributions of *R* and *F* were more balanced (0.31 and 0.32), as were the RIF effects (0.05 and 0.08; where only the latter reached significance).

Similarly, when with 2P modeling of receiver operating characteristics (ROCs) we found the baseline contribution of *R* to be relatively weak (Spitzer and Bäuml, 2007) or even practically absent (Spitzer and Bäuml, 2009), RIF selectively decreased the *F* parameter. Dobler and Bäuml (2013) did not report formal 2P analyses, but inspecting their asymmetric and curvilinear ROCs in light of 2P predictions strongly suggests that both *R* and *F* contributed substantially to NRP recognition – and that both 2P-relevant ROC features (slope and curvature) were affected by RIF. Together, the quality of the RIF-impairment in terms of 2P parameters appears determined by the 2P quality of recognition on baseline. Therein, RIF seems capable of impairing both recollection and/or familiarity, provided they contribute substantially to overall baseline memory performance.

## FINDING RIF IN RECOGNITION

Whether and how retrieval-practice affects recognition memory continues to be of theoretical relevance for the potential cause(s) of RIF. Both inhibitory and non-inhibitory (e.g., blocking) accounts have received empirical support (e.g., Raaijmakers and Jakab, 2012; Storm and Levy, 2012), and the gross impairment seen in recall tests may often result from a mixture of mechanisms. Recent reports that RIF might exclusively affect recall-like processes (i.e., recollection; Verde, 2004; Verde and Perfect, 2011) seemed to severely limit the prospect that recognition tests could help to further disambiguate the net effects on the items’ representation *per se* (cf. Hicks and Starns, 2004) and to thereby expose in particular the contribution of inhibitory suppression (Storm and Levy, 2012). The meta-experimental perspective relativizes this limitation by illustrating how baseline levels – which can largely be experimentally controlled – may determine the quantity and quality of RIF in recognition, and hence its potential diagnosticity for inhibitory as opposed to non-inhibitory mechanisms of forgetting.

At least provisionally, the entirety of the surveyed data appears coherently accommodated by a simple signal detection framework, in terms of a proportional decrement of the affected items’ memory strength. In this light, the past decade has brought accumulating evidence that RIF, unlike many other types of forgetting, can affect recognition memory, and the impairments therein might go beyond a mere mimicry of recall effects (e.g., Grundgeiger, 2013). With respect to the precise qualitative nature of these impairments, the present inventory stresses memory strength as one central factor for increasing the insight from future studies, including the weight of potentially negative results, above what could at this time be inferred already from inspection of baseline levels.

## Conflict of Interest Statement

The author declares that the research was conducted in the absence of any commercial or financial relationships that could be construed as a potential conflict of interest.

## Footnotes

1 The authors reported RIF to be absent also after median-splitting the participant group in *11b* according to performance levels. However, the split was *post hoc* based on the joint variability in *d*′_NRP_ and *d*′_RP-_ (pooled), rendering interpretation of the resultant RIF pattern (*d*′_NRP_ - *d*′_RP-_) difficult. Also, performance in both split-groups (*d*′ = 0.25 and 0.87) fell short of the levels for which robust RIF effects might be expected based on the present analyses (cf. **Figure 1**, *red*).

2 In studies *4a*, *10*, *12*, *15*, *17*, and *20*, matched control materials for RP+ and RP- (NRP+ and NRP-) were used to assess RIF impairments and benefits, respectively. For conditions *2b*-*c*, no RP+ data are available due to design particularities.
